# Which Way Is Down? Positional Distortion in the Tilt Illusion

**DOI:** 10.1371/journal.pone.0110729

**Published:** 2014-10-24

**Authors:** Alessandro Tomassini, Joshua Adam Solomon, Michael John Morgan

**Affiliations:** 1 Sobell Department of Motor Neuroscience and Movement Disorders, Institute of Neurology, University College London, London, United Kingdom; 2 Optometry Division, Applied Vision Research Centre, City University London, London, United Kingdom; 3 Max Planck Institute for Neurological Research, Cologne, Germany; Scientific Institute Foundation Santa Lucia, Italy

## Abstract

Contextual information can have a huge impact on our sensory experience. The tilt illusion is a classic example of contextual influence exerted by an oriented surround on a target's perceived orientation. Traditionally, the tilt illusion has been described as the outcome of inhibition between cortical neurons with adjacent receptive fields and a similar preference for orientation. An alternative explanation is that tilted contexts could produce a re-calibration of the subjective frame of reference. Although the distinction is subtle, only the latter model makes clear predictions for unoriented stimuli. In the present study, we tested one such prediction by asking four naive subjects to estimate three positions (4, 6, and 8 o'clock) on an imaginary clock face within a tilted surround. To indicate their estimates, they used either an unoriented dot or a line segment, with one endpoint at fixation in the middle of the surround. The surround's tilt was randomly chosen from a set of orientations (±75°, ±65°, ±55°, ±45°, ±35°, ±25°, ±15°, ±5° with respect to vertical) across trials. Our results showed systematic biases consistent with the tilt illusion in both conditions. Biases were largest when observers attempted to estimate the 4 and 8 o'clock positions, but there was no significant difference between data gathered with the dot and data gathered with the line segment. A control experiment confirmed that biases were better accounted for by a local coordinate shift than to torsional eye movements induced by the tilted context. This finding supports the idea that tilted contexts distort perceived positions as well as perceived orientations and cannot be readily explained by lateral interactions between orientation selective cells in V1.

## Introduction

**Figure 1:**
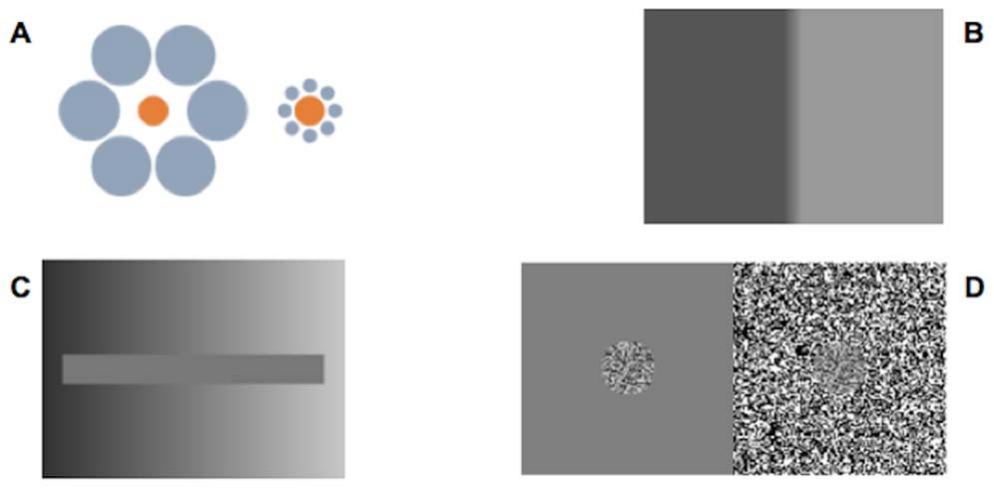
Examples of contextual effects in vision. **Ebbinghaus illusion:** although the two orange circles are exactly the same size, the one on the left appears smaller by virtue of the size of the surrounding circles (Ebbinghaus, 1897). **b) Mach bands:** illusory dark or light stripes are perceived next to the boundary between two regions of an image with different lightness gradients (Mach, 1865). **c) Brightness contrast:** the left end of the horizontal bar appears to be brighter than the right one, depending on the brightness of the surround. In fact, the bar is just one color (Heiring, 1878). **d) Contrast contrast:** a low contrast texture surrounded by an uniform background seems to have higher contrast than the same one but surrounded by a high-contrast texture (Chubb et al., 1989).

**Figure 2:**
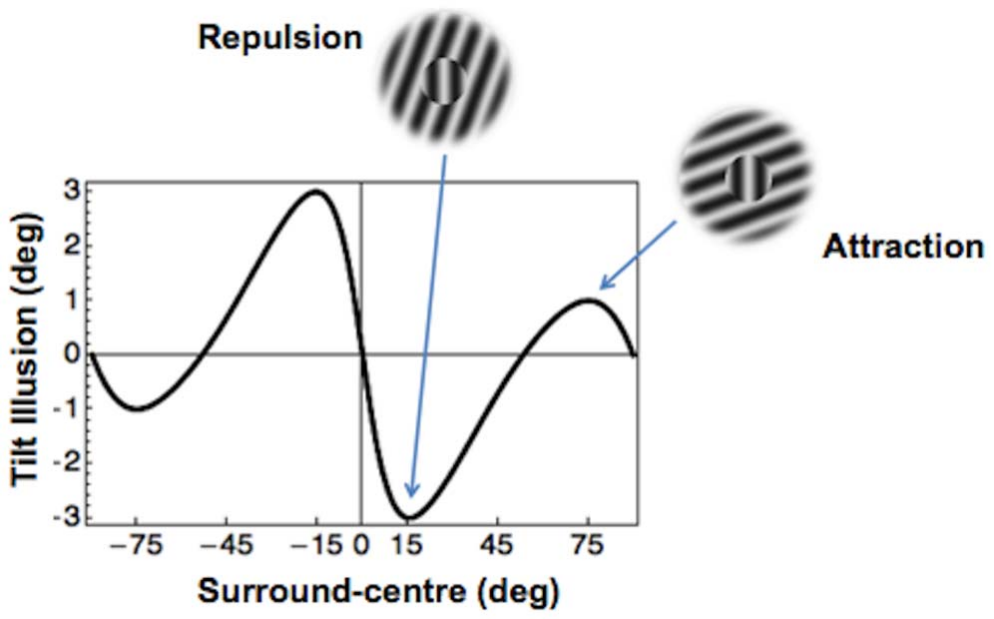
Angular function of the tilt illusion. The plot shows the bias magnitude as a function of the angle difference between surround and target orientations. When a vertically oriented grating is surrounded by a context tilted 15° away (top inset), the visual system exaggerates the difference between their orientations giving rise to the phenomenological repulsion of the vertical stimulus from the surround orientation. For surround-centre angles larger than 60° the illusion is inverted so that the vertical stimulus appears attracted toward to the surround's orientation.

In an informationally redundant visual environment, inhomogeneities (i.e. novelty) provide the most valuable information. The visual system has been shaped by natural selection to extract such inhomogeneites and to discount absolute magnitudes [1]. In doing so, contextual information is not neglected but used to maximize the inhomogeneity's prominence [2], [3]. As an example, think of how small your car appears when surrounded by bigger ones and vice versa (a practical instance of Ebbinghaus Illusion, [4], see Figure 1). Therefore the processing of an input depends strongly on its context. Psychophysical evidence for contextual effects is particularly conspicuous in vision (Figure 1) and encompasses Mach bands [5], [6], brightness contrast [7], [8], and contrast-contrast [9]. In the present work we focused on the tilt illusion, a striking example of contextual influence exerted by an oriented surround on a target's perceived orientation. When a vertically oriented grating (the test stimulus) is surrounded by a context tilted about 15° away, the visual system systematically overestimates the difference between their orientations [10] giving rise to an apparent repulsion (see Figure 2).

One kind of explanation attributes the tilt illusion to mutual inhibition between cortical neurons with adjacent receptive fields and similar preference for orientation. As a consequence, lateral inhibition causes a repulsive shift in neuronal tunings away from the surround's orientation [11], [12], [13]. An alternative to the lateral inhibition mechanism is Gibson's normalization hypothesis. Gibson originally noticed that slightly tilted lines appear to become less tilted over time [14]. He inferred that “we carry around with us our own visual reference-axes with respect to which a line may be seen as upright or tilted”, a “sense of visual direction” [14]. He also proposed that the “visual reference-axes” were not hardwired and could rotate towards the visual context.

Psychophysical evidence in support of Gibsonian normalization comes from studies documenting effects similar to the tilt illusion, but induced by a surrounding square frame on a vertical line: a phenomenon known as the rod-and-frame illusion [15], [16]. Given its global character, the rod-and-frame illusion cannot be explained by local interactions between V1 cells. Instead, it could be understood in terms of a rotation of the visual co-ordinates system inducing a relative distorted perception of the central line [17].

**Figure 3:**
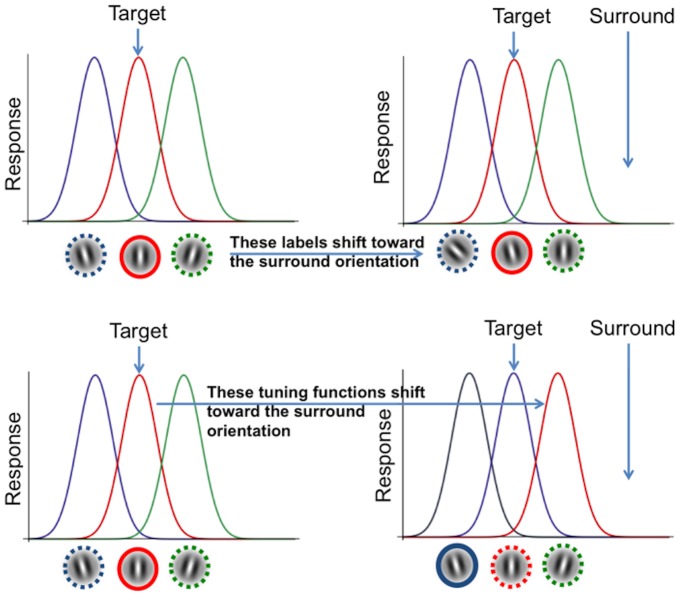
Labels and tuning shift models of contextual influence. **Left: tuning functions** of cortical orientation detectors. When a vertical target is presented alone the vertically selective neurons (red curve) responds strongly (solid circle) giving rise to the perception of a vertical stimulus (red circled perceptual label). **Upper row: label shift model.** Perceptual labels (oriented Gabor patches) shift towards units aligned with the visual context so that vertical orientations are perceived as tilted away. **Lower row: tuning shift model.** Tuning curves are attracted by the surround orientation causing a re-calibration of the vertical towards the surround orientation. Since the perceptual label stay put, the vertical stimulus will excite units labelled as tilted away from vertical, giving rise to a repulsive illusion.

One functional account of Gibson's explanation invokes the shift of the perceptual labels of cardinal orientations, towards cells aligned with the visual context so that physical cardinal orientations are experienced as shifted away. A formally equivalent alternative is the one in which orientation preferences are attracted by the surround orientation while the perceptual labels stay put [18] (see Figure 3). Support for this idea has been provided by a study on motion processing by cortical area MT in macaques [19], [20]. Gibson's normalization could also be expressed in Bayesian inferential terms where the cardinal axes represent priors and these priors can be updated by the statistics of the input [21].

Gibsonian normalization might be the by-product of the ongoing re-calibration of positional relationships, so as to be consistent with the re-mapping of orientation preferences. Whenever the local context is perceived as less oblique, then positional estimates (like “top” and “bottom”) in that region will be shifted accordingly. Such idea sides with recent evidence showing that the oblique effect, (the lower acuity for oblique contours compared to cardinal contours), is affected by both the tilt of head and visual context. This evidence suggests that orientations might be encoded in a multimodal reference frame, which integrates vestibular and peripheral visual information [22].

Although the difference between lateral inhibition and Gibsonian normalization might appear subtle, only the latter can explain the effect of oriented contexts on the perceived positions of isotropic (i.e. non oriented) stimuli.

## Main Experiment

**Figure 4:**
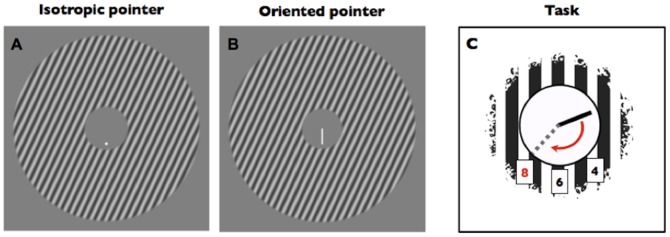
Example stimuli and procedure for the main experiment. **a–b) Stimuli.** On each trial the surround's orientation was randomly drawn from the set *θ* ∈ {±75, ±65, ±55, ±45, ±35, ±25, ±15, ±5}, and the phase was randomised. In the example shown above, the surrounds are tilted 25° clockwise with respect to vertical. On average, our observers showed systematic biases consistent with the tilt illusion in both the conditions. **c) Cartoon of the task**. Observers were requested to align the pointer to one of three possible positions (indicated in red in the figure) on an imaginary clock face by pressing either the left or right arrow key. The trial was terminated by the observer pressing the space bar.

In this first experiment we measured biases in estimating one of three positions (4, 6 and 8 o'clock) on an imaginary clock face within an annularly windowed grating (Figure 4). To indicate their estimates, observers adjusted either the position of a dot or the orientation of a line segment having one endpoint fixed in the middle of the annulus. Since the magnitude of the tilt illusion depends on angular contrast, the surround's tilt was randomly selected across trials from a set of possible orientations. To reiterate, if the tilt illusion were mediated by early interactions between orientation detectors then we would expect it to affect the apparent orientation of the line segment; not the apparent position of the isotropic dot.

### Methods

#### Observers

Four students took part to the experiment, (2 women and 2 men) aged between 23 and 28 years old and with corrected-to-normal vision. They were naïve to the purpose of the experiment.

#### Ethics statement

All participants provided written informed consent. All protocols have been previously approved by the School of Health Sciences Research Ethics Committee (SHS REC) at City University.

#### Apparatus

For this and the subsequent experiment, stimuli were presented using Matlab and the Psychtoolbox routines [23], [24], on a 20-inches calibrated LCD display controlled by an Apple iMac via an ATI Radeon HD 26000 PRO card having 8-bit gray-scale resolution. Each pixel subtended approximately 0.02° of visual angle, at the viewing distance of 60 cm. Observations were carried out in an artificially lighted room. Data analysis was conducted using Mathematica and Psychometrica [25].

#### Stimuli

At a viewing distance of 60 cm, the outer and inner annulus diameters subtended 10° and 2° of visual angle, respectively. The sinusoidal grating had a spatial frequency of 1.9 c/deg, a spatial phase randomly chosen from the interval (−π, +π) a mean luminance of 111 cd/m^2^ and Michelson contrast of 0.99. Both outer and inner annulus borders were smoothed via a raised cosine filter subtending 0.13° of visual angle. On each trial the grating's orientation was drawn from the set {±75, ±65, ±55, ±45, ±35, ±25, ±15, ±5}.

The line segments' width and length were 0.16° and 1.5° respectively, while the dot had an angular subtense of 0.26°. Pointers had random color polarity (black or white), were separated from surround's inner border by a 0.5° gap, and both had their contours smoothed through a raised cosine envelope as to avoid aliasing artifacts.

#### Procedure

An annularly windowed grating was centered on fixation. The lower hemicycle in its inner aperture contained either a dot or a line segment (see Figure 4). On each trial, a number corresponding to of one of three possible positions (4, 6 and 8 o'clock) was showed on the upper part of the display and observers adjusted the dot or the lower endpoint of the segment from its random starting position (from 0° to 360°) to their desired one by pressing the left and right arrow keys. Observers reported satisfaction with their adjustment and readiness for the next trial by pressing the space bar. Each session consisted of 768 trials blocked by pointer type (i.e. dot or segment) with random order between subjects. Target positions were randomly interleaved inside each block.

### Results

In order to deal with the inter-subject variability associated with low precision for oblique locations [26] we defined biases as deviations from each observer's subjective value of the target position. Subjective values for a given location were obtained by averaging the responses over all surround orientations. Consequently, if our observers had shown a constant response bias, say to align the pointer more clockwise than the target's apparent position, then this bias would not be confused with the effect of the surround's orientation. Of course, any response bias that changed with surround orientation would be impossible to divorce from perceptually elicited biases.

The range of surround orientations was centered on the vertical orientation. Consequently, whereas the 6 o'clock reference had an equal range (75 degrees) of annulus tilts on both its sides, the 4 and 8 o'clock references did not. With these references, we considered only those sides with a complete range of surround orientations, and plotted all the data as clockwise of vertical.

**Figure 5:**
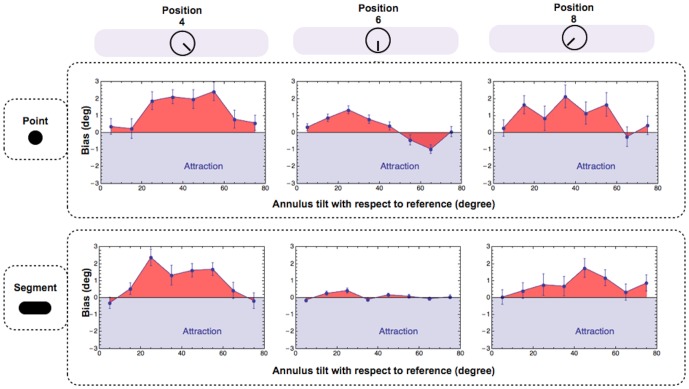
The effect of pointer type and position on alignment biases. Average unsigned alignment biases segregated on the basis of pointer and position. Positive biases with the segment are consistent with the direct tilt illusion. Positive biases with the point are in the same direction. Estimates of the 6 o'clock position were both more precise and more accurate than estimates of the 4 and 8 o'clock positions. Biases were just as large (if not even larger) when observers indicated the target position with an isotropic dot. In all plots error bars contain 2 SEs.

Each point in Figure 5 shows the effect of the surround on the average alignment bias of our four observers, segregated on the basis of the adopted pointer and target position. Error bars contain two standard errors (SEs).The data points that fall in the unshaded regions of this figure indicate a tendency to align the pointer further away from the contextual orientation (analogous to repulsion in the direct tilt illusion).

**Figure 6:**
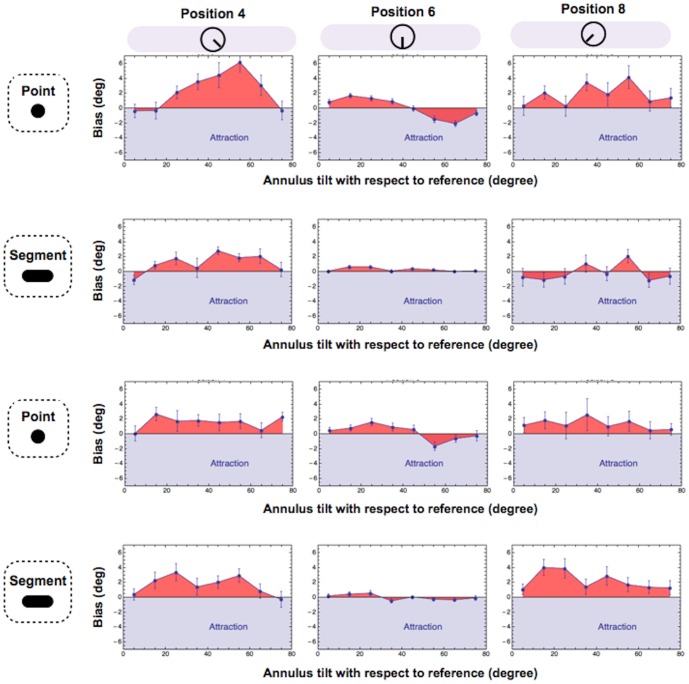
The effect of pointer type and position: individual data. Average alignment biases for each subject, segregated on the basis of pointer and position. Plots show the data from four subjects (2 rows for subject) Also the individual data show an higher precision for estimates of the 6 o'clock position. Biases for dot and segments look pretty much similar. In all plots error bars indicate 95% confidence intervals.

As expected, estimates of the 6 o'clock position were both more precise and more accurate than those of the 4 and 8 o'clock positions. The largest biases were found when the grating's orientation was 25° clockwise and anti-clockwise of the target position. These results are consistent with previous studies of the tilt illusions [27] showing largest repulsive biases for test-surround angles between 5° and 30°. Systematic biases with the unoriented dot were as large (if not even larger) as those with the line segment. The significance of biases for both pointers was confirmed by statistical analysis (one-tailed t-test against zero across positions and orientations; dot: t(3)  =  5.409, p  =  0.006; segment: t(3)  =  2.583, p  =  0.04). The same pattern of results can be appreciated in Figure 6, in which the data have been further segregated on the basis of observer. The statistical significance of individual biases is reported on Table 1.

**Table 1:** Individual biases.

Subject	Pointer	Location	t	df	Sig. (1-tailed)	Mean Difference	95% CI of the Difference	
							Lower	Upper
1	Dot	4	2.909	6	.014	2.659	.422	4.896
		6	.275	6	.396	.150	−1.188	1.488
		8	3.268	6	.009	1.869	.469	3.268
	Segment	4	2.534	6	.022	1.243	.042	2.444
		6	2.877	6	.014	.289	.043	.535
		8	−.278	6	.395	−.129	−1.268	1.009
2	Dot	4	4.301	6	.003	1.431	.617	2.245
		6	.703	6	.250	.290	−.719	1.299
		8	5.496	6	.001	1.414	.784	2.044
	Segment	4	4.606	6	.002	1.891	.886	2.896
		6	.119	6	.450	.018	−.356	.393
		8	4.960	6	.001	2.332	1.181	3.482
3	Dot	4	1.201	6	.135	.472	−.489	1.433
		6	1.083	6	.160	.309	−.390	1.009
		8	1.866	6	.056	.949	−.295	2.193
	Segment	4	.951	6	.185	.297	−.467	1.061
		6	−.167	6	.435	−.027	−.428	.373
		8	2.509	6	.023	.609	.014	1.203
4	Dot	4	2.414	6	.026	1.382	−.018	2.784
		6	3.094	6	.011	.570	.119	1.022
		8	.130	6	.450	.061	−1.092	1.215
	Segment	4	1.906	6	.053	1.214	−.344	2.772
		6	2.325	6	.030	.086	−.004	.177
		8	1.012	6	.175	.317	−.450	1.085

One-tailed t-test against zero was adopted to assess the statistical significance (p <0.05) of individual biases on the basis of the pointer stimulus and location.

To further assess the significance of our results we performed a repeated measures ANOVA (full factorial design, two pointers x three target positions x 8 surround's orientations; Table 2) on the means of each observer. Annulus' tilt showed a significant effect on the induced bias [F(7, 21)  =  4.685, p  =  0.003]. A significant two-way interaction was observed only for pointer x annulus tilt [F(7, 21)  =  2.578, p  =  0.044] however, Bonferroni post hoc analysis failed to indicate any significant (p <0.05) difference. There were neither significant main effect of adopted pointer [F(1,3)  =  1.005, p  =  0.39], nor of position [F(2,6)  =  4.28, p  =  0.07].

**Table 2:** Repeated measures ANOVA.

Source	Type III Sum of Squares	df	Mean Square	F	Sig.
Pointer	3.334	1	3.334	1.005	.390
Error(pointer)	9.950	3	3.317		
Location	33.379	2	16.689	4.280	.070
Error(location)	23.399	6	3.900		
Orientation	48.189	7	6.884	4.685	.003
Error(orientation)	30.860	21	1.470		
Pointer * location	.337	2	.168	.070	.933
Error(pointer*location)	14.443	6	2.407		
Pointer * orientation	7.732	7	1.105	2.578	.044
Error(pointer*orientation)	8.998	21	.428		
Location * orientation	26.083	14	1.863	1.491	.157
Error(location*orientation)	52.472	42	1.249		
Pointer * location * orientation	10.442	14	.746	1.183	.323
Error(pointer*location*orientation)	26.476	42	.630		

Full factorial 2 (stimuli) x 3 (reference positions) x 8 (surround's orientations) ANOVA with repeated measured confirms no significant difference between biases induced on a segment or on a dot. The only significant effect is exerted by annulus' orientation and by the interaction of annulus' tilt with pointer type.

## Control Experiment

The first experiment revealed a systematic bias consistent with the tilt illusion in both conditions. However, it might be argued that the effect could be attributed to torsional eye movements induced by the tilted context [28]. Indeed, experimental evidence indicates that a stationary tilted visual stimulus can induce the illusion of self-tilt in the opposite direction of the stimulus [29], [30], [31], [32]. The most relevant of these studies with regard to our question [32] has revealed visually induced torsional eye movements towards the stimulus' orientation (although as small as 0.5°; [16], [32]). If the subjective vertical were encoded only by virtue of retinal coordinates, then when the eyes' vertical meridians were rotated relative to the physical vertical, the subjective vertical would rotate accordingly. Thus in our second experiment we used two annular gratings, in mirror symmetry, flashed on each side of fixation, and asked our observers to judge the positions of dots presented within their apertures. In this way, it seems unlikely that torsional eye movements would affect the judgment, but local coordinate shifts would.

Here and in experiment 1, focus is put only on the so called “direct” or repulsive effect of the tilt illusion which occurs when the target-surround angle is between 5° and 35°. We did not investigate its attractive counterpart (i.e. the indirect effect) as it is assumed to be mediated by high-level (i.e. non V1) processes [33] and is therefore not critical in testing the lateral inhibition model.

### Methods

#### Observers

Four students, different from those who took part to the first experiment, (1 woman and 3 men) aged between 23 and 28 years old and with corrected-to-normal vision. Also they were naïve to the purpose of the experiment.

Apparatus was identical to the main experiment.

#### Ethics statement

All participants provided written informed consent. Experimental protocols conformed to the guidelines of the Declaration of Helsinki and were approved by City University's research ethics committee.

#### Stimuli

Annuli were the same as in experiment 1 except for the dimensions having an outer and inner outer diameter subtending 7° and 2° of visual angle, respectively, at a viewing distance of 57 cm. Dots had random color polarity (black or white). Stimuli on both the sides were mirror symmetrical across the vertical meridian: this arrangement was chosen to discourage torsional eye movements induced by the tilted context.

#### Procedure

**Figure 7:**
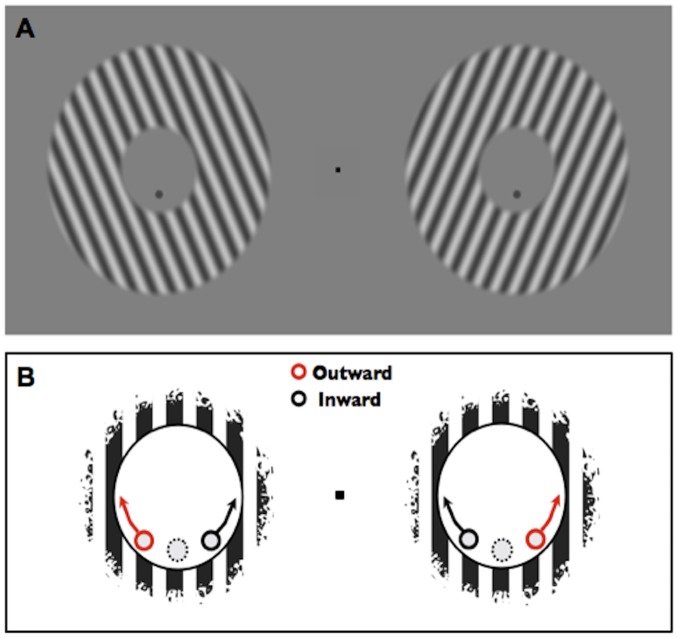
Example stimuli and procedure for the control experiment. **a) Stimuli.** Two sinusoidal grating annuli were flashed for 100 ms on the right and left side of a central fixation point. The angle of the dots positioned inside each annulus varied symmetrically among trials in accordance with a staircase adaptive algorithm. **b) Cartoon of the task.** Observers had to press the left or right arrow keys to report whether the two dots' positions appeared inward or outward with respect to the central fixation point.

Two sinusoidal grating annuli were flashed for 100 ms on the right and left side of a central fixation point. A dot was positioned along the lower hemicycle of each annulus inner aperture (see Figure 7) and its angle (with respect to the vertical) was adjusted by one of 20 interleaved Gaussian Quest Staircases, two (to get a full psychometric curve we estimated 16% and 84% thresholds) for each possible surround's orientation: {±35, ±30, ±25, ±20, ±15}. We asked our observers to fixate a central black square (0.18°) and to press the left or right arrow keys to report whether the two dots' positions appeared “inward” or “outward”, with respect to the central fixation point. No feedback was given and fixation was not controlled in any way.

Each observer performed four blocks of 400 trials each.

### Results

**Figure 8:**
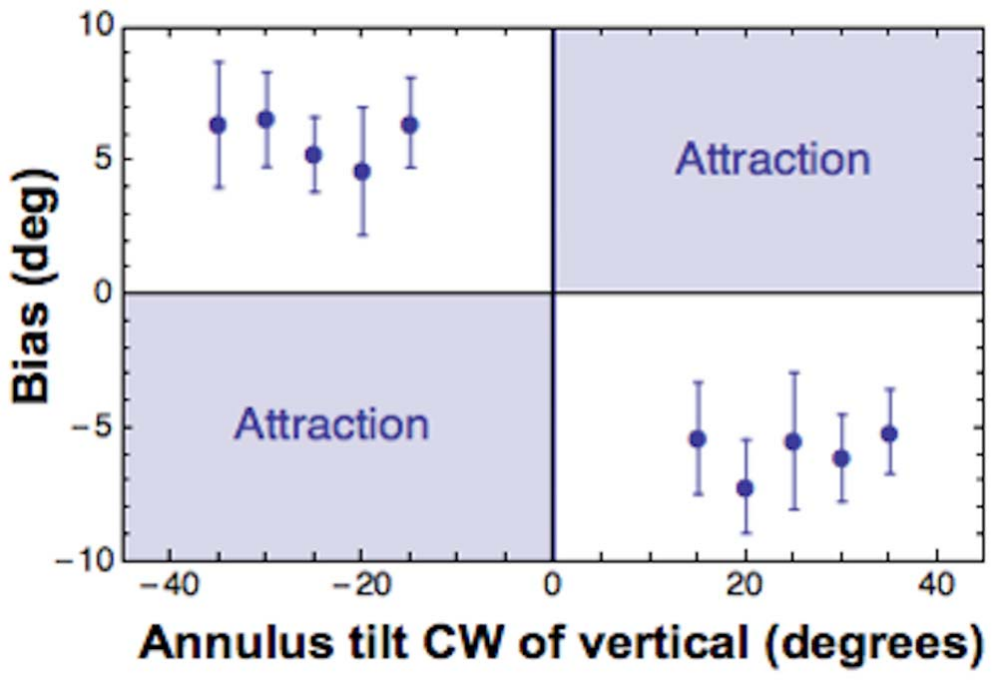
The effect of contextual orientation on the induced position bias. Point of subjective verticality estimated for each annulus' tilt and collapsed over observers. This configuration produces even larger biases, consistent with previous studies of the tilt illusion. In all plots error bars contains 2 MSE.

**Figure 9:**
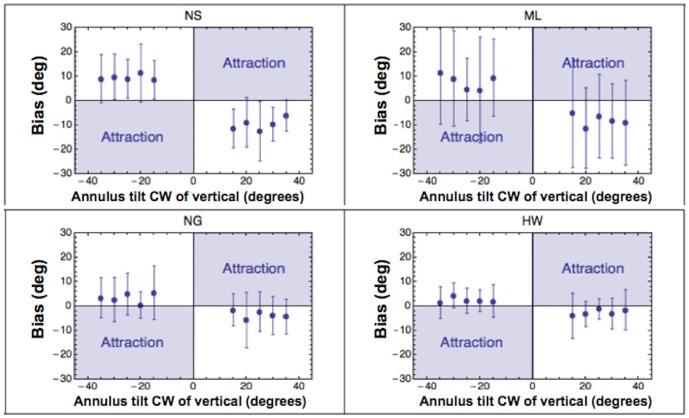
The effect of contextual orientation on position bias, individual data. Point of subjective verticality estimated for each annulus' tilt for each observer. Aside from intersubjective differences in magnitude, the pattern of position biases is fairly consistent across subjects. In all plots error bars contains 95% confidence intervals.

Points of subjective verticality (PSV) were estimated for each annulus's orientation, corresponding to the angle at which dot is perceived aligned to annulus's median axis (i.e. vertical in the orientation domain). Those values indicate the amount of spatial distortion, or bias, induced by contextual orientation in the perception of dot's position. Although there was some inter-subject variability, all of our data showed a systematic distortion of dot's perceived position. When collapsing biases across subjects this trend is clearly visible and the significance of the effect is confirmed by statistical analysis [t-test against zero; t(9)  =  23.14, p <0.001], (see Figure 8 and Figure 9 for collapsed and individual data respectively). This result is consistent with the idea that tilt illusion (i.e. distortion in perceived tilt) might be caused by a rotation of perceived locations (i.e. rotation of reference - axes) rather than orientation per se.

## Discussion

Our results show a systematic distortion of the perceived location of a dot within an oriented grating, consistent with the direction of the tilt illusion. The bias measured with non-oriented targets mirrors and sometimes exceeds the ones observed when a segment is adopted as a test stimulus. Lateral inhibition models of tilt illusion rely on early mechanisms selective to orientation. The lateral inhibition exerted by neurons activated by an oriented surround would produce a repulsive shift in the population response giving rise to the phenomenological repulsion of a vertical test from the surround's orientation. Since dots have no orientation, lateral inhibition models fail to account for our observed biases. Our control experiment shows that this distortion should not be attributed to torsional eye movements either [34], as a similar bias was obtained when the same stimuli were presented yoked in mirror symmetry (i.e. with opposite orientations) beside fixation.

The data are therefore consistent with a shift (or re-calibration) of the subjective vertical towards the orientation of the surround. Such mechanism could allow the visual system to re-calibrate and thus match changes in the physical world [35]. One potential further benefit of this re-calibration is dynamic range optimization. By centering the neural activity to a dominant property of the visual scene, redundancy between the responses of neurons would be diminished, maximizing the bandwidth available for the transmission of novel information about the stimulus [36], [37].

Many different sensorial dimensions are organized in oppositional scales, so that they have a norm or null point. In the orientation domain the vertical axis might be considered as a neutral point between clockwise and anti-clockwise orientations. Perhaps such a norm is not hard-wired but constantly extracted from the environmental stimulation. A similar extraction has been inferred from studies of body roll. For tilt angles under 60° human subjects overestimate the actual body tilt (this has been dubbed the E-effect; [38]). That is, the subjective visual vertical shifts away from the body axis [39], [40], [41], so that the actual gravitational vertical appears to be deflected towards the body, just as in the tilt illusion's indirect effect.

Adaptive re-calibration is consistent with a Bayesian account of the subjective frame. In this framework, prior knowledge is used to resolve uncertainty in the interpretation of noisy information. The estimation of the visual vertical could be biased by the “a priori” probability that surrounding gratings are vertical. Since we usually experience a visually upright world it might be that our visual system assumes that the subjective vertical is most likely to be aligned with the vertical axis of the visual scene.

Although it is still unknown where in the brain gravitational and visual information are integrated into a world-centered frame of reference, it might be that such integration could start early in the visual pathway. Indeed, single-cell studies on monkeys and cats have reported body tilt-dependent changes of orientation selectivity on V1 and V2 neurons [42], [43]. The results of these studies agree with psychophysical work pointing to a role of gravity in defining the subjective vertical for the oblique effect [44], [45], [46], [47]. Specifically, in tilted conditions the subjective visual vertical is the orientation reproduced with highest precision, while the oblique effect observed in upright posture is abolished [47]. Hence, the oblique effect might be mapped in a subjective gravitational reference frame centered on the subjective visual vertical. Moreover, the subjective vertical is not only affected by the tilt of head but also by the tilt of visual oriented cues, coherently with the idea of a multimodal reference frame for the encoding of orientations [48].

Our interpretation is consistent with psychophysical data reported by Aubert (1861, [49]) showing that a physically vertical line of light in a dark room appeared tilted away when observed from a roll position exceeding 60° [50], [51], [39], [52]. The line appeared vertical only after being rotated by 45 degrees (Aubert effect, [49]). We could draw a parallel between the spatial distortion perceived in the Aubert effect and the overestimation of the body roll reported in the E-effect, by assuming that both are due to a rotation of the subjective vertical towards and away the body axis, respectively.

The major implication of our work is that an oriented surround can elicit a positional distortion on an isotropic stimulus. Gibsonian normalization is consistent with some plasticity in either the labeling or the preferences of orientation-selective neurons. Our results suggest that this plasticity extends to neurons that encode the positional relationships between isotropic stimuli.
